# Retracted: Extreme Anemia (Hemoglobin 1.8 g/dL) Secondary to Abnormal Uterine Bleeding

**DOI:** 10.1155/2019/6025938

**Published:** 2019-04-24

**Authors:** Case Reports in Obstetrics and Gynecology

At the request of the authors, the article titled “Extreme Anemia (Hemoglobin 1.8 g/dL) Secondary to Abnormal Uterine Bleeding” [1] has been retracted. The article was found to contain a substantial amount of material from the following published article, cited as [8]:

R. E. Schmitt and C. J. Buckley II, “Extreme Anemia (Hemoglobin 1.8 G/Dl) Secondary to Colon Cancer,” Baylor University Medical Center Proceedings, 29:4, 393-394, 2016. [2]

The article was also found to contain a substantial amount of material, without citation, from the following published article:

B. Ma, “Excessive Uterine Bleeding in a Non-Compliant Patient with Profound Hypothyroidism: A Case Report and Review of the Literatures,” Journal of Medical Cases, North America, 2016. [3]
